# Anti-PD-1 therapy in advanced sarcomas: is cutaneous primary site a stronger predictor of response than histologic subtype?

**DOI:** 10.1007/s00262-023-03387-6

**Published:** 2023-03-13

**Authors:** Ruoyu Miao, Jennifer Swank, Dan Melzer, Steven Ludlow, Leah Clark, Molly Finger, Damon R. Reed, Mihaela Druta, Andrew S. Brohl

**Affiliations:** 1 Hematology and Medical Oncology, Moffitt Cancer Center, Tampa, FL USA; 2 Pharmacy Department, Moffitt Cancer Center, Tampa, FL USA; 3 Sarcoma Department, Moffitt Cancer Center, Tampa, FL 33612 USA; 4 Department of Individualized Cancer Management, Moffitt Cancer Center, FL Tampa, USA

**Keywords:** Sarcoma, Immune checkpoint inhibitors, Programmed cell death 1 receptor, Skin neoplasms

## Abstract

**Background:**

Immune checkpoint inhibitors (ICIs) have shown modest antitumor activity in unselected advanced sarcomas. Histology driven approach to patient selection is the current standard for off-label anti-programmed cell death 1 (PD1) immunotherapy use.

**Methods:**

We retrospectively reviewed the clinical characteristics and outcomes of patients with advanced sarcoma who were treated with off label anti-PD1 immunotherapy at our center.

**Results:**

A total of 84 patients with 25 histological subtypes were included. Nineteen patients (23%) had a cutaneous primary tumor site. Eighteen patients (21%) were classified as having clinical benefit, including 1 patient with complete response, 14 with partial response, and 3 with stable disease lasting over 6 months with previously progressive disease. Cutaneous primary site location was associated with higher clinical benefit rate (58% vs*.* 11%, p < 0.001), longer median PFS (8.6 vs*.* 2.5 months, *p* = 0.003) and OS (19.0 vs. 9.2 months, *p* = 0.011), compared to non-cutaneous primary. Patients with histological subtypes that pembrolizumab is indicated per current National Comprehensive Cancer Network guidelines had modestly higher rate of clinical benefit versus other histologies, however, the difference was statistically insignificant (29% vs. 15%, *p* = 0.182) and no statistically significant difference in PFS or OS was observed between these groups. Immune-related adverse events were more frequently seen among patients with clinical benefit (72% vs. 35%, *p* = 0.007).

**Conclusions:**

Anti-PD1-based immunotherapy is highly efficacious in advanced sarcomas of cutaneous primary site. Cutaneous primary site location is a stronger predictor of ICI response than histologic subtype and should be accounted for in treatment guidelines and clinical trial design.

**Supplementary Information:**

The online version contains supplementary material available at 10.1007/s00262-023-03387-6.

## Introduction

Immune checkpoint inhibitors (ICIs) targeting programmed cell death 1 (PD-1) or PD-ligand-1 (PD-L1), have led to remarkable outcomes in many cancer types. Anti-PD1 therapy has been particularly effective in virtually every cutaneous malignancy in which this therapy class has been evaluated formally, likely in part due to the stigmata of a high ultraviolet (UV)-mutational burden that is common across these cancer types.[1–3] In contrast, reports from clinical trials with pembrolizumab or nivolumab as either monotherapy or in combination with ipilimumab have shown only modest antitumor activity in unselected advanced sarcomas, with response rates of roughly 10–20% when including all histological subtypes.[4–7]. Responses to ICIs are noted to be histology-dependent, with higher response rates reported in undifferentiated pleomorphic sarcoma (UPS),[6] alveolar soft part sarcoma (ASPS),[8, 9] cutaneous angiosarcoma,[10] and Kaposi sarcoma,[11, 12] etc. However, because of the diversity and rarity of sarcoma, most clinical trials have varied histology specific representation and small sample sizes.[13] Current NCCN guidelines[14, 15] reflect a histology-driven approach to patient selection for anti-PD1 immunotherapy in sarcoma, with pembrolizumab being listed as an off-label consideration for later lines of therapy for selected sarcoma histologic subtypes including myxofibrosarcoma, UPS, cutaneous angiosarcoma and undifferentiated sarcomas and as a preferred therapy option for ASPS. As in all solid tumors, high mutational burden (≥ 10 mutations/Mb) is also considered by guidelines as an indication to consider anti-PD1 therapy for patient that are refractory to other standard treatments.

In this study, we reviewed the clinical characteristics and outcomes of patients who were treated with ICI for advanced sarcoma at our institution and sought to explore the factors that are associated with clinical benefit from ICI. We hypothesized that a cutaneous primary site location would predict for higher chance of ICI benefit.

## Materials and methods

We retrospectively reviewed all patients with advanced sarcoma who were treated with off-label pembrolizumab or nivolumab at Moffitt Cancer Center (MCC). We did not include patients treated with this agent class in the context of a clinical trial. All patients who received at least one dose of ICI were included in this study. This study was reviewed and approved by the Institutional Review Board at MCC.

Treatment response was assessed according to Response Evaluation Criteria in Solid Tumors (RECIST) 1.1. Patients with best response of complete response (CR), partial response (PR) or stable disease (SD) for at least 6 months in previously progressive disease were considered having clinical benefit from ICI. Overall survival (OS) was defined as the time interval from the initiation of ICI to death from any cause. Progression-free survival (PFS) was defined as the time interval from the initiation of ICI to disease progression or death. Adverse events were graded based on Common Terminology Criteria for Adverse Events (CTCAE) Version 5.0.

### Statistical analysis

Statistical significance between groups was analyzed using the chi-square tests or Fisher’s exact test for categorical variables and the Student’s t-test or Mann–Whitney U non-parametric test for continuous variables. The estimated OS and PFS were derived using the Kaplan–Meier method and compared by the Mantel-Cox log-rank test. The statistical analyses were performed using IBM SPSS Statistics for Windows, version 28.0 (IBM Corp, Armonk, NY). Kaplan–Meier survival curves were generated in R (version 4.2.1; www.r-project.org), using the ‘survminer’ package and the ‘ggsurvplot’ function. All reported *p* values were two-sided. The level of significance was set at *p* < 0.05.

## Results

### Clinical characteristics

A total number of 88 patients who received pembrolizumab or nivolumab for advanced sarcoma between November 2015 and July 2022 were identified. Four patients were excluded for further analysis due to the lack of adequate information for outcome evaluation, generally due to lack of follow-up and/or transfer of care to an alternate location. The clinical characteristics of the remaining 84 patients are summarized in Table 1.

**Table 1:** Clinical characteristics

		All		No clinical benefit from ICI		Clinical benefit		P value
		84		66	79%	18	21%	
Age	mean (SD)	62.3	(15.8)	60.4	(15.6)	69.4	(14.7)	**0.031**
Gender	Male	52	62%	37	56%	15	83%	0.054
	Female	32	38%	29	44%	3	17%	
ECOG PS	0	15	18%	10	15%	5	29%	0.247
	1	54	66%	43	66%	11	65%	
	2	13	16%	12	18%	1	6%	
Site	Cutaneous/dermal	19	23%	8	12%	11	61%	** < 0.001**
	Other	65	77%	58	88%	7	39%	
ICI-indicated histology by NCCN guideline	Yes^a^	38	45%	27	41%	11	61%	0.182
	No^b^	46	55%	39	59%	7	39%	
Distant metastasis	No	8	10%	4	6%	4	22%	0.061
	Yes	76	90%	62	94%	14	78%	
PD-L1	Negative	19	50%	15	58%	4	33%	0.295
	Positive	19	50%	11	42%	8	67%	
Prior systemic therapy	0	24	29%	15	23%	9	50%	0.067
	1	28	33%	23	35%	5	28%	
	≥ 2	32	38%	28	42%	4	22%	
irAE	No	48	57%	43	65%	5	28%	**0.007**
	Yes	36	43%	23	35%	13	72%	

Bold = p<0.05

ECOG, eastern cooperative oncology group; ICI, immune check point inhibitor; irAE, immune-related adverse events; NA, not available; NCCN, national comprehensive cancer network; PD-L1, programmed death-ligand 1; PS, performance status; SD, standard deviation

^a^undifferentiated pleomorphic sarcoma (19), cutaneous angiosarcoma (12), alveolar soft part sarcoma (5), myxofibrosarcoma (2)

^b^dedifferentiated liposarcoma (9), conventional chondrosarcoma (5), leiomyosarcoma (4), non-cutaneous angiosarcoma (3), osteosarcoma (3), clear cell sarcoma (2), dedifferentiated chondrosarcoma (2), gastrointestinal stromal tumor (2), classic Kaposi sarcoma (2), undifferentiated sarcoma of bone (2), breast sarcoma NOS (1), epithelioid sarcoma (1), GREB-NCOA2 fusion uterine sarcoma (1), fibrosarcoma (1), malignant glomus tumor (1), malignant peripheral nerve sheath tumor (1), myoepithelial carcinoma (1), pleomorphic liposarcoma (1), pleomorphic rhabdomyosarcoma (1), SMARCA4-deficient thoracic sarcoma (1), solitary fibrous tumor (1), synovial sarcoma (1)

There were 52 men and 32 women with a median age of 64 years (range: 17–91 years). Among the 25 histological subtypes of sarcoma included in this study, the most common were UPS (19, including 4 pleomorphic dermal sarcomas [PDS]), angiosarcoma (15, including 12 cutaneous), dedifferentiated liposarcoma (DDLS, 9), ASPS (5), and conventional chondrosarcoma (5). The primary tumor of 19 patients (23%) was cutaneous/dermal sarcoma, including angiosarcoma (12), UPS/PDS (4), classic Kaposi sarcoma (CKS, 2), and a dermal fibrosarcoma (1). Two patients had radiation-associated tumors, one non-cutaneous angiosarcoma and one osteosarcoma. At the time of ICI initiation, 76 patients (90%) had distant metastasis, and the remaining 8 (10%) had locally or regionally advanced disease. PD-L1 IHC status was known in 38 patients, 19 of whom (50%) were considered as PD-L1 positive. Ten patients had information on MSI status, only one was MSI-high with a known diagnosis of Lynch syndrome. TMB was assessed in 5 patients, only one had high TMB over 10 mutations per megabase (54 muts/Mb).

The median number of prior systemic therapy was 1 (range: 0–13). ICI was given as first line in 24 patients (29%), second line in 28 patients (33%), and third line and above in 32 patients (38%). The majority of patients received single agent pembrolizumab (75) while pembrolizumab was combined with eribulin (two DDLSs and one leiomyosarcoma), Lenvatinib (two leiomyosarcomas), and axitinib (one ASPS). For the remaining 3 patients, nivolumab was given alone (2, DDLS and gastrointestinal stromal tumor [GIST]) or in combination with ipilimumab (1, GIST). The median number of ICI administered was 5 doses (range: 1–38).

### Outcome

**Fig. 1:**
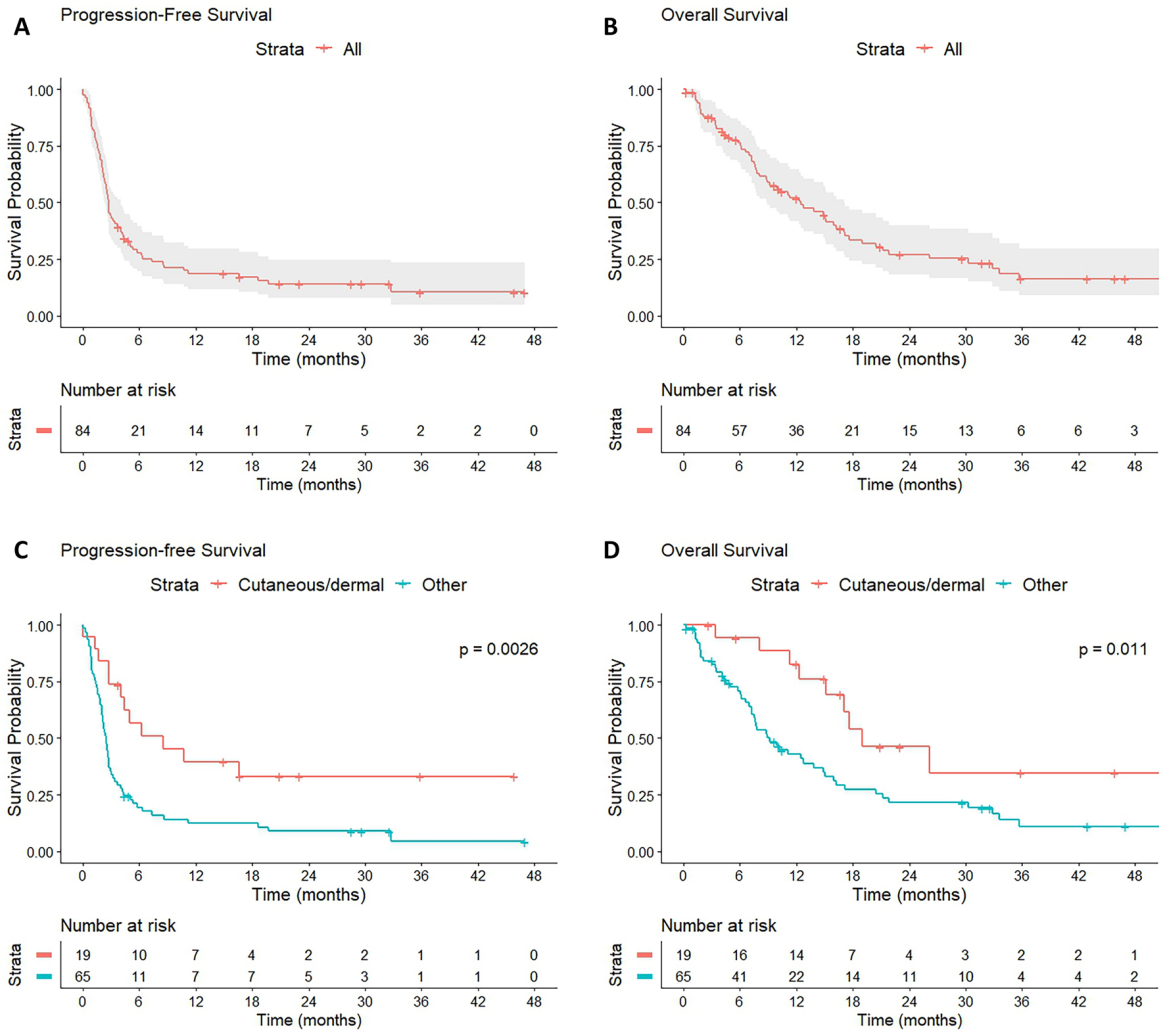
Kaplan–Meier survival analysis of patients treated with immune checkpoint inhibitor. Kaplan–Meier curves of progression-free survival (**A**) and overall survival (**B**) of all patients and progression-free survival (**C**) and overall survival (**D**) of patients stratified by primary tumor site

Best response was CR in one patient with cutaneous angiosarcoma (1%), PR in 14 patients (17%, 2 cutaneous KS, 2 cutaneous UPS/PDS and 2 UPS of other sites, 2 cutaneous angiosarcomas and 1 angiosarcoma of other site, 1 ASPS, 1 DDLS, 1 dermal fibrosarcoma, 1 pleomorphic rhabdomyosarcoma, and 1 SMARCA4-deficient thoracic sarcoma), SD in 13 patients (15%), and PD in 56 patients (67%). The median PFS and OS was 2.7 months (95% confident interval [CI]: 2.4–3.1 months) and 12.5 months (95% CI: 7.7–17.3 months), respectively (Table S1, Fig. 1). irAEs of any grade were seen in 36 patients (43%), 11 of them (13%) had grade 3/4 irAEs.

Eighteen patients (21%) were classified as having clinical benefit, including the 1 CR, 14 PRs, and 3 patients with SD > 6 months with previously progressive disease (2 cutaneous UPS/PDS, 1 cutaneous angiosarcoma). Of the 3 patients with SD classified as having clinical benefit, all were progressing at treatment initiation, all experienced clinical tumor regression not meeting the RECIST threshold for PR, and duration of response was 8 months in one patient and is ongoing at 16 + and 45 + months in the other two. All 18 patients classified as having clinical benefit received single agent anti-PD1. The median PFS and OS among the patients with clinical benefit were 32.7 months (no estimation on 95% CI) and not reached, respectively, whereas the median PFS and OS among those without clinical benefit were 2.2 months (95% CI: 1.8–2.7 months) and 8.8 months (95% CI: 6.5–11.2 months), respectively (Table S1). irAEs were more frequently seen among patients with clinical benefit (72% vs. 35%, *p* = 0.007, Table 1).

There were more patients with cutaneous sarcoma who experienced clinical benefit compared to those with primary tumor of other sites (11/19 [58%] vs. 7/65 [11%], *p* < 0.001, Table 1). The median PFS (8.6 months vs. 2.5 months, *p* = 0.003) and OS (19.0 months vs. 9.2 months, *p* = 0.011) were significantly longer among patients with cutaneous sarcoma than those with non-cutaneous primary (Table S1, Fig. 1).

Patients with the histological subtypes that pembrolizumab may be indicated per current NCCN guideline, i.e., UPS, cutaneous angiosarcoma, ASPS and myxofibrosarcoma in this study, had trend towards higher rate of ICI clinical benefit, however, the difference was not statistically significant (11/38 [29%] vs. 7/46 [15%], *p* = 0.182, Table 1). Similarly, there was non-statistically significant tendency of patients to have a greater chance of clinical benefit for those with locoregional disease only, positive PD-L1, or no or fewer prior systemic therapies. No statistically significant difference in PFS or OS was observed based on above factors (Table S1). Better Eastern Cooperative Oncology Group (ECOG) performance status (PS) at the initiation of ICI was associated with longer OS (16.3 months in PS 0 vs. 12.8 months in PS 1 vs. 4.5 months in PS 2, *p* = 0.024).

## Discussion

We report our institutional experience with off-label anti-PD1-based immunotherapy use in advanced cases of sarcoma. In 84 patients representing 25 different sarcoma histologic subtypes, we report an overall clinical benefit rate of 21% in our cohort. While we noted a modest enrichment for treatment benefit if applying a histology-based, NCCN guideline-like patient selection criteria, our most striking observation was that cutaneous primary site location was highly predictive of ICI benefit, with a clinical benefit rate of 58% (11 of 19) based on this single clinical factor.

The benefit of ICI therapy in 21% of our overall cohort is congruent with prior reports,[4, 6, 7] though slightly on the high end of expected benefit for single-agent anti-PD1 in unselected sarcoma patients. This is very likely explained by a biased patient selection towards off-label anti-PD1 use in sarcoma histologies that are felt to be more immunotherapy responsive based on prior reports.[6, 8–12] Notably, almost half of our cohort (38 of 84, 45%) is represented by NCCN-guideline suggested, “anti-PD1 responsive” histologic subtypes.

Most notably, we observe a signal for oncologic benefit for anti-PD1 therapy in sarcomas from a cutaneous primary site, with an overall 58% clinical benefit rate in this subset of patients across several sarcoma histologic subtypes. This observation is concordant with prior reports in angiosarcoma, [10, 16, 17] but to our knowledge has not been as well appreciated in other sarcoma histologic types such as undifferentiated sarcomas [18]. Presumably, higher mutational burden due to the genomic stigmata of UV mutagenesis as has been reported in several cutaneous sarcoma subtypes [18, 19] might in part explain the higher response rate of cutaneous sarcomas to this drug class. The observation of high ICI benefit rate related to cutaneous primary site has particular importance for UPS, a sarcoma subtype in which emphasis has been placed for checkpoint inhibitor clinical trials and clinical practice. All four of the cutaneous UPS (a.k.a. pleomorphic dermal sarcomas) in our cohort experienced clinical benefit from anti-PD1 therapy, as opposed to only 2 of 15 (13%) UPS tumors from other primary locations.

Our study has several limitations. Due to the rarity of sarcomas, the sample size of our cohort is small, although it represents a relatively large single institutional study. The retrospective design also makes it difficult to draw definitive conclusions. In addition, the low percentage of NGS and PD-L1 IHC tests performed in our study limited our ability to evaluate TMB and PD-L1 expression as potential predictive biomarkers.

Our findings have important implications for anti-PD1 therapy and anti-PD1 based clinical trials in sarcoma patients. First, the magnitude of overall benefit of anti-PD1 therapy in cutaneous sarcomas that we observed compares favorably to other cutaneous malignancies such as melanoma in which this agent class has clearly become a preferred first line treatment option. Further, it is well-known that deferring checkpoint inhibitor therapy to later lines of therapy in other immunotherapy-responsive cutaneous malignancies is detrimental to outcomes for the general patient population, even when the alternative first line therapy has significant disease efficacy such as BRAF/MEK inhibitor therapy in melanoma [20] or cytotoxic chemotherapy in Merkel cell carcinoma [2, 21]. It would therefore stand to reason that anti-PD1 based immunotherapy should be evaluated as first line therapy for unresectable or metastatic cutaneous sarcomas, even when alternative options with anti-tumor efficacy exist, e.g. taxane-based cytotoxic chemotherapy for cutaneous angiosarcoma. Second, for ICI clinical trial design in sarcomas, we believe that it is imperative that cutaneous primary site location be considered at a minimum as a predefined stratification variable for randomization and for outcome reporting. Currently, we observe that this approach is being increasingly adopted for angiosarcoma but has not been widely considered for “undifferentiated sarcoma” trials. Specifically for UPS/undifferentiated sarcoma, we also believe it essential for any ICI trial to attempt NGS to help rule out a UV-mutation signature, the finding of which could only be present if the primary site was dermal or if the tumor was a misdiagnosed spindle cell variant of melanoma.

In summary, we review our institutional experience with off-label anti-PD1 immunotherapy in advanced sarcoma patients. We observe striking efficacy of this drug class in sarcomas with cutaneous primary site location, rivaling that of other cutaneous malignancies in which checkpoint inhibitor therapy is the well-established first line therapy. If confirmed in follow-up reports, our findings have profound implications for patient selection for anti-PD1 therapy and for ICI clinical trial design for sarcoma histologic subtypes that can originate in the dermis, such as undifferentiated pleomorphic sarcomas.

### Supplementary Information

Below is the link to the electronic supplementary material.

Supplementary file1 (DOCX 15 KB)

## Abbreviations

ASPS: Alveolar soft part sarcoma
CKS: Classic Kaposi sarcoma
CTCAE: Common Terminology Criteria for Adverse Events
CR: Complete response
DDLS: Dedifferentiated liposarcoma
ECOG: Eastern Cooperative Oncology Group
GIST: Gastrointestinal stromal tumor
ICI: Immune checkpoint inhibitor
irAEs: Immune-related adverse events
IHC: Immunohistochemistry
MSI: Microsatellite instability
MCC: Moffitt Cancer Center
NCCN: National Comprehensive Cancer Network
NGS: Next-generation sequencing
OS: Overall survival
PR: Partial response
PS: Performance status
PDS: Pleomorphic dermal sarcomas
PD-1: Programmed cell death 1
PD-L1: Programmed cell death ligand-1
PFS: Progression-free survival
PD: Progressive disease
RECIST: Response Evaluation Criteria in Solid Tumors
SD: Stable disease
TMB: Tumor mutational burden
UV: Ultraviolet
UPS: Undifferentiated pleomorphic sarcoma
WHO: World Health Organization
