# Supplementary material for: Anti-PD-1 therapy in advanced sarcomas: is cutaneous primary site a stronger predictor of response than histologic subtype?
Source: Cancer Immunol Immunother. 2023 Mar 13;72(7):2521–7. doi: 10.1007/s00262-023-03387-6 (PMC10264480; doi:10.1007/s00262-023-03387-6)
Supplement: Supplementary file 1 — Supplementary file1 (DOCX 15 KB) [file 262_2023_3387_MOESM1_ESM.docx]

Table 2. Median overall survival and progression-free survival estimates.

|  | Overall survival (months) | | | Progression-free survival (months) | | |
| --- | --- | --- | --- | --- | --- | --- |
| Variable | Median | 95% CI | Log-rank P | Median | 95% CI | Log-rank P |
| Overall | 12.5 | 7.7-17.3 |  | 2.7 | 2.4-3.1 |  |
| Gender |  |  | 0.249 |  |  | 0.082 |
| Male | 12.5 | 6.7-18.3 |  | 2.8 | 2.4-3.2 |  |
| Female | 11.3 | 3.9-18.8 |  | 2.4 | 1.6-3.1 |  |
| ECOG PS |  |  | **0.024** |  |  | 0.050 |
| 0 | 16.3 | 10.6-21.9 |  | 2.6 | 0-6.1 |  |
| 1 | 12.8 | 7.5-18.1 |  | 2.8 | 2.2-3.4 |  |
| 2 | 4.5 | 0.9-8.2 |  | 1.2 | 0-2.7 |  |
| Site |  |  | **0.011** |  |  | **0.003** |
| Cutaneous/dermal | 19.0 | 10.2-27.8 |  | 8.6 | 0.8-16.3 |  |
| Other | 9.2 | 6.1-12.2 |  | 2.5 | 2.0-3.0 |  |
| ICI-indicated histology by NCCN guideline |  |  | 0.347 |  |  | 0.105 |
| Yes | 15.2 | 12.4-17.9 |  | 3.3 | 1.4-5.1 |  |
| No | 8.9 | 5.8-11.9 |  | 2.4 | 1.8-2.9 |  |
| Distant metastasis |  |  | 0.527 |  |  | 0.373 |
| No | 20.5 | 7.9-33.1 |  | 2.1 | 0-10.4 |  |
| Yes | 12.3 | 8.2-16.4 |  | 2.7 | 2.5-3.0 |  |
| PD-L1 |  |  | 0.666 |  |  | 0.256 |
| Negative | 13.8 | 5.6-22.1 |  | 2.7 | 0.8-4.6 |  |
| Positive | 32.9 | 2.9-62.9 |  | 3.3 | 0-7.1 |  |
| Prior systemic therapy |  |  | 0.316 |  |  | 0.348 |
| 0 | 33.6 | 3.5-63.6 |  | 3.3 | 0.0-7.8 |  |
| 1 | 11.3 | 7.4-15.3 |  | 2.6 | 2.1-3.1 |  |
| ≥2 | 11.1 | 3.1-19.1 |  | 2.7 | 2.3-3.0 |  |
| ICI Response |  |  | **<0.001** |  |  | **<0.001** |
| Clinical benefit | 8.8 | 6.5-11.2 |  | 2.2 | 1.8-2.7 |  |
| No clinical benefit | Not reached |  |  | 32.7 |  |  |

ECOG, Eastern Cooperative Oncology Group. ICI, immune check point inhibitor. NCCN, National Comprehensive Cancer Network. PD-L1, Programmed death-ligand 1. PS, performance status.
